# Role of PPARs in Radiation-Induced Brain Injury

**DOI:** 10.1155/2010/234975

**Published:** 2009-09-17

**Authors:** Sriram Ramanan, Weiling Zhao, David R. Riddle, Mike E. Robbins

**Affiliations:** ^1^Department of Cancer Biology, Comprehensive Cancer Center, Wake Forest University School of Medicine, Winston-Salem, NC 27157, USA; ^2^Brain Tumor Center of Excellence, Comprehensive Cancer Center, Wake Forest University School of Medicine, Winston-Salem, NC 27157, USA; ^3^Department of Radiation Oncology, Comprehensive Cancer Center, Wake Forest University School of Medicine, Winston-Salem, NC 27157, USA; ^4^Department of Neurobiology and Anatomy, Comprehensive Cancer Center, Wake Forest University School of Medicine, Winston-Salem, NC 27157, USA

## Abstract

Whole-brain irradiation (WBI) represents the primary mode of treatment for brain metastases; about 200 000 patients receive WBI each year in the USA. Up to 50% of adult and 100% of pediatric brain cancer patients who survive >6 months post-WBI will suffer from a progressive, cognitive impairment. At present, there are no proven long-term treatments or preventive strategies for this significant radiation-induced late effect. Recent studies suggest that the pathogenesis of radiation-induced brain injury involves WBI-mediated increases in oxidative stress and/or inflammatory responses in the brain. Therefore, anti-inflammatory strategies can be employed to modulate radiation-induced brain injury. Peroxisomal proliferator-activated receptors (PPARs) are ligand-activated transcription factors that belong to the steroid/thyroid hormone nuclear receptor superfamily. Although traditionally known to play a role in metabolism, increasing evidence suggests a role for PPARs in regulating the response to inflammation and oxidative injury. PPAR agonists have been shown to cross the blood-brain barrier and confer neuroprotection in animal models of CNS disorders such as stroke, multiple sclerosis and Parkinson's disease. However, the role of PPARs in radiation-induced brain injury is unclear. In this manuscript, we review the current knowledge and the emerging insights about the role of PPARs in modulating radiation-induced brain injury.

## 1. Introduction

PPARs are ligand-activated transcription factors that belong to the steroid/thyroid hormone superfamily of nuclear receptors [1, 2]. To date, three PPAR isotypes have been identified—PPAR*α* (NR1C1), PPAR*β* (NR1C2 or PPAR*δ*), and PPAR*γ* (NR1C3) [3]. Each is encoded by a separate gene, and each has a unique tissue distribution pattern [4, 5]. PPARs regulate gene transcription by heterodimerizing with the retinoid X receptor (RXR) and binding to specific consensus sequences (termed PPAR response elements, PPREs) in the enhancer regions of genes [6]. PPREs consist of a direct repeat (DR) of the nuclear receptor hexameric recognition sequence AGGTCA separated by one or two nucleotides (DR-1 or DR-2) [6]. The protein structure of the PPAR isotypes reveals two well-characterized domains— a highly conserved DNA binding domain and a ligand-binding domain (LBD) that is less well conserved across the subtypes. Variation in the sequence of amino acids that line the ligand-binding pocket is a major determinant of ligand isotype specificity [7, 8]. In the absence of ligand binding, PPAR-RXR heterodimers are bound to corepressor proteins such as HDACs and N-CoRs that maintain the chromatin in the condensed state and inhibit the transcriptional apparatus from assembling. Upon ligand binding, PPARs undergo a conformational change that leads to dissociation of the corepressor proteins. Subsequently, the ligand-bound PPARs complex with coactivator proteins such as p300 leading to nucleosome remodeling and transcriptional preinitiation complex assembly on target gene promoters [7]. The transcriptional response is strongly influenced by the structure of the promoter and the expression levels of coactivators and corepressors in a given cell-type [9].

## 2. Tissue Distribution and Physiological Role of PPARs

PPAR*α* is predominantly expressed in tissues that catabolize high amounts of fatty acids such as the liver, skeletal muscle, and heart and regulates many metabolic pathways, including activation of fatty acid *β*-oxidation and apolipoprotein expression [10]. Natural ligands such as eicosanoids, mono- and polyunsaturated fatty acids and long-chain fatty acyl-CoenzymeA can bind and activate PPAR*α* [7]. Hypolipidemic fibrate drugs that are routinely prescribed to patients for lowering triglyceride and cholesterol levels have been demonstrated to be synthetic ligands of PPAR*α* [11, 12]. 

 PPAR*γ* is most abundantly expressed in fat cells, the large intestine, and cells of the monocyte lineage. It is primarily involved in the general transcriptional control of adipocyte differentiation, immune responses, and glucose homeostasis [13, 14]. PPAR*γ* exists as two distinct forms, *γ*1 and *γ*2, which arise by differential transcription start sites and alternative splicing [15]. Whereas PPAR*γ*1 is low in most tissues, PPAR*γ*2 is fat-selective and is expressed at very high levels in adipose tissue [14]. PPAR*γ* is bound and activated by several naturally occurring compounds, such as the eicosanoids 9- and 13-hydroxyoctadecadienoic acids [8]. More recently, a type of nitrated lipids known as nitroalkenes has been demonstrated to be potent, endogenous ligands of PPAR*γ* [16]. In addition, several high-affinity synthetic PPAR*γ* agonists have been synthesized, including the thiazolidinedione (TZD) class of compounds [17], which are used clinically as insulin sensitizers in patients with type 2 diabetes [18], and certain nonsteroidal antiinflammatory drugs [19]. 

 Unlike the PPAR*α* and PPAR*γ* isotypes, the expression of PPAR*δ* appears to be ubiquitous. Ligands of PPAR*δ* include fatty acids such as bromopalmitate [20] and the prostanoid prostacyclin PGI_2_ [21]. Studies suggest key roles for PPAR*δ* in proliferation [22], differentiation, and survival as well as in embryonic development and fatty acid *β*-oxidation in skeletal muscles and adipose tissues [22]. More recently, PPAR*δ* agonists have been shown to enhance oligodendrocyte maturation and differentiation [23]. Mice that are knocked-out for PPAR*δ* have altered myelination in the corpus callosum suggesting a role for PPAR*δ* in myelination [24].

## 3. PPARs in the Central Nervous System

All PPAR isotypes have been identified in the rodent brain, and their expression has been shown to peak between day 13.5 and 18.5 of gestation. The degree of expression and specific localization varies among the receptors. PPAR*δ* appears to be expressed ubiquitously in all regions of the brain [4], primarily in oligodendrocytes and neurons [25] and to a lesser extent in astrocytes [4]. In the spinal cord white matter, expression is localized to oligodendrocytes [26]. PPAR*α* expression in the brain appears limited to the olfactory bulbs, hippocampus, and cerebellum, primarily in cerebellar granule neurons (CGNs) [5] and astrocytes. In the spinal cord white matter, expression of PPAR*α* is localized to astrocytes [26]. PPAR*γ* has been observed, albeit at relatively low levels, in the hippocampus, cerebellum as well as in cortical astrocytes and CGN [4]. All three PPAR isoforms are expressed in the microglia [27]. 

 All PPAR isoforms have been proposed to play an important role in the developing and adult brain [28]. PPAR*α* has been shown to play a major role in acetylcholine biosynthesis, excitatory amino acid neurotransmission, and defense against oxidative stress [29]. PPAR*γ* is coexpressed along with dopominergic receptors in several regions of the brain suggesting that it could regulate the action of dopamine on gene transcription [29]. PPAR*δ*-mediated transcriptional upregulation of Acyl-CoA synthetase 2 mediates fatty acid utilization and plays an important role in brain lipid metabolism. Experiments using neurosphere cultures derived from mouse neural stem cells isolated from the subventricular zone (SVZ) have demonstrated that PPARs regulate the proliferation and fate of these cells [30, 31].

## 4. Anti-Inflammatory/Neuroprotective Role of PPARs in Neurodegenerative Disorders

In addition to their well-known functions on cellular metabolism, PPARs have been shown to play a major role in inflammation. The anti-inflammatory functions of PPARs in several peripheral tissues have been reviewed elsewhere [2, 32] and beyond the scope of this review. With reference to the CNS, several studies have documented the anti-inflammatory and neuroprotective effects of PPAR ligands in a number of neuropathological conditions [27, 33]. 

 In vitro models of Alzheimer's disease (AD), PPAR*γ* agonists inhibited the neuronal death induced by the amyloid-*β* (A*β*) peptide by inhibiting the microglial and monocytic proinflammatory response and astrocytic proliferation [34]*.* In vivo, oral administration of the PPAR*γ* agonist pioglitazone reduced glial activation and the accumulation of A*β*-positive plaques in the hippocampus and cortex [35, 36]. In a clinical trial involving 500 AD patients, a significant improvement in cognitive function was observed following treatment with the PPAR*γ* agonist, rosiglitazone for 6 months [37]. 

 In a mouse model of Parkinson's disease, oral administration of pioglitazone inhibited the glial activation induced by the neurotoxin 1-methyl-4-phenyl-1, 2, 3, 6-tetrahydropyridine (MPTP) and prevented the loss of dopominergic neurons in the substantia nigra pars compacta. Mechanistically, the neuronal death was prevented by (i) inhibiting the nuclear translocation of the redox-regulated proinflammatory transcription factor NF-*κ*B subunit p65 and (ii) preventing the subsequent induction of inducible nitric oxide synthase (iNOS) gene [38]. Similar protective effects on dopominergic neurons were demonstrated following administration of the PPAR*α* agonist fenofibrate [39]. 

 PPAR agonists have also been shown to reduce the severity of cerebral ischemic injury in rodents. Oral administration of PPAR*α* agonists decreased the incidence of stroke in apolipoprotein-E deficient mice and reduced the cerebral infarct volume in wild-type mice following transient middle cerebral artery occlusion (MCAO) [40, 41]. These effects were associated with decreased oxidative stress and adhesion molecule expression in the brain [40]. Other studies have reported that administration of PPAR*α* agonists either prior to cerebral ischemia or during the reperfusion period can also have a neuroprotective effect [42, 43]. Likewise, administration of the PPAR*γ* agonists troglitazone or pioglitazone, or the PPAR*δ* agonists L-165041 or GW501516, prior to or during transient MCAO reduces the infarct volume [44–46]. 

 In the mouse model of multiple sclerosis (MS), experimental autoimmune encephalomyelitis (EAE), PPAR agonists have been shown to delay the onset and reduce the severity of the disease. PPAR*α* agonists inhibited the proliferation of CD4^+^ T-cells and shifted their differentiation pattern from the proinflammatory Th1-type to the anti-inflammatory Th2-type cells [47]. In addition, oral administration of the PPAR*α* agonists, gemfibrozil and fenofibrate, alleviated the clinical symptoms of EAE [47, 48]. Administering the PPAR*δ* agonist GW0742 in the mouse diet during the peak of EAE can improve clinical recovery, partly by reducing lymphocyte infiltration into the CNS and by decreasing resident glial activation [49]. Numerous research studies have demonstrated the anti-inflammatory and neuroprotective role of PPAR*γ* agonists in reducing the neurological symptoms of chronic progressive and relapsing forms of EAE [50–52]. 

 Since resident glial cell inflammation and immune cell infiltration into the brain are considered hallmarks of several neuroinflammatory disorders, numerous research groups have hypothesized that the neuroprotective effects of PPAR agonists might result, in part, from inhibition of proinflammatory responses during the CNS pathology. Consistent with this hypothesis, PPAR agonists have been shown to inhibit myelin oligodendrocyte glycoprotein-, cytokine-, and lipopolysaccharide-induced increases in proinflammatory mediators such as tumor necrosis factor alpha (TNF*α*), members of the interleukin (IL) family such as interleukin 1 beta (IL-1*β*) and IL-12, cyclooxygenase-2 (Cox-2), iNOS, and interferon gamma (IFN-*γ*) as well as the expression of adhesion molecules such as monocyte chemoattractant protein-1 (MCP-1) in the astrocytes, microglia, and T-cells in vitro [53–57]. 

 Taken together, these data suggest that PPAR agonists show promise as efficacious anti-inflammatory agents in ameliorating the clinical symptoms and disease severity of a variety of CNS pathologies.

## 5. Whole-Brain Irradiation and Radiation-Induced Brain Injury

Ongoing advancements in cancer treatment and healthcare have led to an increase in the long-term survivors of cancer; >67% of adult and >75% of pediatric cancer patients will survive longer than 5 years after initial diagnosis. As a result, late effects remain a significant risk for these ~11 million cancer survivors. Given the increasing population of long-term survivors, the need to mitigate or treat late effects has emerged as a primary area of research in radiation biology [58, 59]. 

 The total dose of radiation therapy that can be administered safely to the brains of patients presenting with primary or metastatic brain tumors is limited by the risk of normal brain morbidity. The need to both understand and minimize the side effects of brain irradiation is intensified by the ever-increasing number of patients with secondary brain metastases (mets) that require treatment with partial or whole-brain irradiation (WBI). Of the ~1 500 000 new cancer patients diagnosed in 2008 [60], up to 30% will develop brain mets [61, 62], making this the 2nd most common site of metastatic cancer, the most common neurological manifestation of cancer, and a cancer problem more common than newly diagnosed lung, breast, and prostate cancer combined. The annual incidence in the US appears to be increasing, as a result of an aging population, better treatment of systemic cancer, and the application of superior imaging techniques such as magnetic resonance imaging (MRI) to detect smaller and micrometastatic lesions in asymptomatic patients [63]. WBI is the primary mode of treatment for brain mets; up to 170 000 individuals will ultimately be treated with large field or WBI each year in the USA. Over half of these patients will survive long enough to develop radiation-induced brain injury, including cognitive impairment. Presently, there are no successful long-term treatments or effective preventive strategies for radiation-induced brain injury [64]. 

 Classically, based on the time of expression, radiation-induced brain injury has been subdivided into acute, subacute (early delayed), and late delayed responses [65]. Acute injury is expressed days to weeks after irradiation and is often characterized by drowsiness, vomiting, headache, and nausea. This type of injury can be treated with corticosteroids and is fairly uncommon under current radiotherapy regimens [65, 66]. Early delayed injury typically occurs from 1- to 6- months postradiation therapy and can involve transient demyelination, short-term memory loss, fatigability, and somnolence. While both these early injuries can result in severe reactions, they are normally reversible and resolve spontaneously. In sharp contrast, late delayed effects, distinguished by demyelination, vascular abnormalities and ultimate radionecrosis of the white matter are observed >6 months postirradiation and are usually irreversible and progressive [67]. Intellectual deterioration is also seen in patients receiving brain irradiation [68]. Data suggest that 20%–50% of brain tumor patients who are long-term survivors suffer from progressive cognitive dysfunction, ranging from mild lassitude to significant memory loss and severe dementia [69–72]. More importantly, in both clinical and preclinical models, the cognitive impairment has been shown to occur in the absence of gross histopathological and radiographic alterations [73–75].

## 6. Mechanisms of Radiation-Induced Brain Injury: Role of Oxidative Stress, Neuroinflammation, and Impaired Neurogenesis

Conventionally, late effects were thought to be the consequence of a reduction in the number of surviving clonogens of either the parenchymal or the vascular target cell populations [65, 76]. Radiation-induced late normal tissue injury was considered to be inevitable, progressive, and untreatable. However, recent data suggest that this view is over-simplistic and that radiation-injury involves complex, intercellular, and intracellular interactions between various cell types [59, 65, 77–79] (in the brain these include astrocytes, microglia, neurons, etc.) within an organ and can be modulated [59]. In general, irradiating late responding normal tissues is hypothesized to activate autocrine and paracrine signal transduction events that initiate downstream reactive processes marked by a persistent oxidative stress and cytokine production ultimately contributing to tissue injury. Although the cellular, molecular, and biochemical mechanisms of radiation-induced brain injury are ill-defined, several studies lend support to the hypothesis that such an injury is driven, in part, via increased oxidative stress and/or inflammation [73, 80–83]. 

 Irradiating one hemisphere of postnatal day 8 rats or of postnatal day 10 mice with a single dose of 4–12 Gy of 4 MV X-rays led to time-dependent increases in nitrotyrosine, a marker for protein nitrosylation, in the SVZ and the granule cell layer (GCL) of the hippocampus 2–12 hours postirradiation [84]. WBI of the mouse brain with a single dose of 6 Gy led to a significant increase in markers of lipid peroxidation and DNA oxidation such as 4-hydroxynonenal and 8-hydroxy-2′-deoxyguanosine, respectively, 1-month postirradiation in the dentate gyrus (DG), and hilus of the hippocampus [85]. In addition to their direct damaging effects on the DNA, lipids, and proteins, reactive oxygen species can act as second messengers to initiate neuroinflammation [86]. 

 Although the brain traditionally has been considered to be immune-privileged, it is widely accepted now that the brain does exhibit inflammation [87]. An acute molecular response characterized by increased expression of inflammatory cytokines/mediators such as TNF*α*, IL-1*β*, intracellular adhesion molecule-1 (ICAM-1), Cox-2, and activation of transcription factors such as NF-*κ*B and activator protein-1 (AP-1) is observed within hours of irradiating the rodent brain [88–90]. In addition, a chronic elevation of TNF*α* has been observed in the mouse brain up to 6-month postirradiation [91]. 

 More recently, the detrimental effect of WBI on ongoing hippocampal neurogenesis and the associated neuroinflammatory response characterized by activated microglia have been proposed as a key mechanism of radiation-induced cognitive impairment [73, 92]. The hippocampus is situated in the medial-temporal lobe and is one of two regions in the mammalian brain where active neurogenesis occurs throughout adulthood. Neurogenesis is a complex multistep process which starts with the proliferation of the neural precursor cells residing in a specialized region called the subgranular zone (SGZ) of the hippocampus, followed by commitment to a neuronal phenotype, physiological, and morphological maturation with the development of synaptic and electrophysiological properties and ending with the integration of a functional neuron into the GCL [93]. Adult neurogenesis has been shown to play an important role in certain types of hippocampal-dependent cognitive function [94]. 

 One of the earliest observations that led to the proposed involvement of the hippocampus in radiation-induced brain injury was that the extent of cognitive impairment experienced by patients receiving radiotherapy correlated with the dose delivered to the medial-temporal lobe [95]. Subsequently, experimental studies have demonstrated that the neural precursor cells in the SGZ are extremely sensitive to radiation [80]. In vitro, irradiation reduces the proliferative capacity of cultured neural precursor cells [96]. In vivo, the ability of these precursor cells to give rise to new neurons in the GCL is significantly ablated by WBI [80, 96]. More importantly, the WBI-induced decrease in neurogenesis is associated with deficits in hippocampal-dependent spatial learning and memory tasks in mice [97–99]. Furthermore, the deleterious effect of WBI on hippocampal neurogenesis was associated with an increase in the number of activated microglia, suggesting an inflammatory response in the brain following irradiation [80, 96, 98]. A role for the brain microenvironment in the ablation of neurogenesis was further supported by the demonstration that neural precursor cells isolated from nonirradiated brains failed to give rise to new neurons when transplanted into irradiated brains [96]. A negative correlation between activated microglia and hippocampal neurogenesis has been demonstrated, suggesting that the WBI-induced neuroinflammatory response could lead to the impaired neurogenesis [80, 96, 100]. Moreover, administration of the anti-inflammatory drug, indomethacin, decreased radiation-induced microglial activation and partially restored neurogenesis [81]. Together, these data suggest that altered neurogenesis as a result of oxidative stress and/or neuroinflammation is one of the mechanisms of radiation-induced brain injury. Thus, anti-inflammatory strategies might be useful in preventing radiation-induced late effects in the brain. 

 An additional and intriguing aspect of inflammation is the putative link between inflammation and impaired PPAR expression. Analysis of gene expression in postmortem brain tissue obtained from AD patients revealed significant decreases in PPAR*α* and PPAR*δ* gene expression, determined using real time quantitative PCR [101]. Preliminary studies from our own laboratory indicate that treating rat brain microvascular endothelial cells with ionizing radiation or hydrogen peroxide leads to a rapid and significant reduction in PPAR*γ* mRNA and protein (*unpublished data*). Similar changes have been observed in vivo; one year after a fractionated dose of 40 Gy *γ*-rays, PPAR*γ* gene expression was markedly lower than that observed in age-matched sham-irradiated rat brains. Although preliminary, these data confirm previous studies in which radiation has been shown to reduce PPAR expression within hours to several days of treatment in the kidney [102] and the colon [103]. 

 Given that several PPAR agonists are potent neuroprotective/anti-inflammatory agents in several neuroinflammatory disorders, we hypothesized that activation of PPARs will ameliorate the WBI-induced brain injury.

## 7. Effect of PPAR Agonists on Radiation-Induced Brain Injury

### 7.1. In Vitro Studies

A growing body of evidence suggests that the microglial proinflammatory response following radiation contributes to the observed radiation-induced late effects. In vitro studies suggest that irradiating microglia leads to a marked increase in expression of proinflammatory genes including TNF*α*, IL-1*β*, IL-6, and Cox-2 [104–106]. Radiation-induced expression of microglial TNF*α* and IL-1*β* has been shown to enhance leukocyte adhesion in the brain, partly via increased expression of ICAM-1 in astrocytes [104]. Cox-2-mediated production of prostaglandin E2, TNF*α*, and IL-1*β* from the conditioned media of irradiated BV-2 cells has been shown to induce astrogliosis [106]. These studies are supported by in vivo experiments in rodents which indicate that brain irradiation leads to a marked increase in microglial activation associated with both a concomitant decrease in neurogenesis in the hippocampus and spatial memory retention deficits as mentioned previously [97–99]. Thus, modulating the microglial proinflammatory response presents a promising approach to ameliorate radiation-induced brain injury. 

 Extending previous findings, we observed that irradiating BV-2 microglial cells led to a significant increase in TNF*α* and IL-1*β* gene expression and Cox-2 protein levels [107]. The promoter regions of TNF*α*, IL-1*β*, and Cox-2 contain numerous transcription factor binding sites including AP-1 and NF-*κ*B and numerous reports suggest that their expression in the microglia is regulated by these transcription factors [108–112] . Consistent with this, marked increases in the DNA binding activity of AP-1 and NF-*κ*B as early as 30-minutes postirradiation were observed in the microglial cells. Moreover, using specific inhibitors of AP-1 and NF-*κ*B, it was demonstrated that the radiation-induced increase in TNF*α* and Cox-2 expression was AP-1 mediated while that of IL-1*β* was mediated by both NF-*κ*B and AP-1. 

 Given the potent anti-inflammatory properties of PPAR*α* ligands in a variety of cell types including microglia [54, 55, 113], we hypothesized that activation of PPAR*α* in the microglia would inhibit the radiation-induced proinflammatory response. Indeed, the radiation-induced increases in TNF*α*, IL-1*β* gene expression, and Cox-2 protein were significantly inhibited by the PPAR*α* agonists, GW7647, and fenofibrate. Mechanistically, PPAR*α* agonists prevented the activation of AP-1 (by inhibiting nuclear c-jun phosphorylation) and NF-*κ*B (by preventing p65 nuclear translocation) following irradiation thereby inhibiting the microglial proinflammatory response [107]. These findings emphasize the pleiotropic effects of PPAR*α* agonists in response to inflammation as they target multiple proinflammatory microglial cytokines that might be involved in the development and progression of radiation-induced brain injury.

### 7.2. In Vivo Studies

The potent in vitro efficacy of PPAR*α* ligands in modulating the radiation-induced microglial proinflammatory response, along with the negative correlation between microglial activation and hippocampal neurogenesis, led to the hypothesis that activation of PPAR*α* in vivo would prevent the detrimental effect of WBI on neurogenesis and inhibit microglial activation (Ramanan et al. *unpublished data*). In this study, wild-type (WT) mice were divided into 4 groups: (1) Sham-irradiation and control diet, (2) Sham-irradiation and fenofibrate (Fen; 0.2% w/w), (3) WBI (delivered as a single dose of 10 Gy *γ*-rays with half the dose (5 Gy) delivered to each side of the head) and control diet, (4) WBI and fenofibrate. For measuring neurogenesis in the DG, all groups of mice received I.P injections of bromodeoxyuridine (BrdU; 50 mg/Kg body weight) to label the surviving neural precursor cells in the SGZ at 1 month post-WBI (as previously reported in [80, 96, 98]). The number of newborn neurons arising out of these surviving cells was assessed 2-month post-WBI by using double immunofluorescence to detect BrdU and NeuN (a neuronal marker). Consistent with previous findings [80, 96], WBI led to a significant decrease in the number of newborn neurons in the DG that was prevented in the irradiated mice that received fenofibrate in their diet. Furthermore, fenofibrate increased the total number of BrdU^+^ cells in the DG of irradiated animals, suggesting that the PPAR*α* agonist promoted the survival of newborn cells following irradiation. 

 For the assessment of neuroinflammation, brains isolated either 1-week or 2-month post-WBI were subjected to staining with anti-CD68 antibody to label activated microglia. Consistent with our hypothesis, fenofibrate inhibited the WBI-induced increase in number of activated microglia at 1-week post-WBI. Therefore, the preservation of hippocampal neurogenesis by fenofibrate is associated with decreased microglial activation following WBI. Moreover, the number of activated microglia returned to control levels by 2 -month post-WBI, the time point at which we observed a significant decrease in the number of newborn neurons. Thus, the radiation-induced neuroinflammatory response characterized by increased microglial activation might be an early event and could be one of the key components driving the detrimental effects of radiation on ongoing hippocampal neurogenesis. 

 Some studies have documented that fenofibrate can act independently of PPAR*α* [48, 114, 115]. To address this issue, the studies above were replicated in PPAR*α* knock-out (KO) mice. The genetic ablation of PPAR*α* prevented the protective effect of fenofibrate following WBI. These findings highlight the critical role played by PPAR*α* in modulating radiation-induced brain injury as well as providing mechanistic insight into the neuroprotective and anti-inflammatory properties of fenofibrate. 

 A striking difference was observed in the response of the microglial cells to WBI between the WT and PPAR*α* KO mice. Whereas the number of activated microglia returned to control levels by 2 -month post-WBI in the WT mice, activated microglia remained significantly elevated in the KO mice. This suggests that the KO mice show a sustained neuroinflammatory response following WBI. Consistent with these data, preliminary findings from our laboratory suggest that the KO mice brains have a sustained increase in NF-*κ*B DNA binding activity up to 24-hour post-WBI. In addition, the SGZs of these mice have a significantly lower level of basal proliferation compared to age-matched WT mice *(unpublished observations*). These findings are not surprising; PPAR*α* KO mice exhibit a prolonged response to inflammatory stimuli such as lipopolysaccharide and leukotriene B4 [116, 117]. In addition, they develop a physiologically aged phenotype earlier in life compared to the WT mice indicating a role for PPAR*α* in maintaining the cellular redox balance [118]. Thus, it is possible that the lack of PPAR*α* enhances the basal level of inflammation and thereby leads to a protracted response to radiation injury. Nevertheless, the experiments using the KO mice served as a reliable experimental control for the off-target effects of fenofibrate and underlined the importance of PPAR*α* in radiation-induced brain injury (Ramanan et al. *unpublished data*). Whether PPAR*γ* and *δ* ligands mediate similar protective effects on hippocampal neurogenesis following WBI is not yet known and is being actively investigated in our laboratory.

## 8. Effect of PPAR Agonists on WBI-Induced Cognitive Impairment

Functionally, radiation-induced brain injury is characterized by a progressive, cognitive impairment that severely compromises the quality of life (QOL) of cancer patients receiving radiotherapy. Given the increasing evidence for a role of oxidative stress/inflammation in radiation-induced brain injury, Zhao et al. tested the hypothesis that the PPAR*γ* agonist pioglitazone (Pio) would ameliorate the severity of radiation-induced cognitive impairment in a well-characterized rat model of fractionated WBI. Young adult male F344 rats were divided into five experimental groups: (1) fractionated WBI; 40 or 45 Gy *γ* rays delivered as eight or nine 5 Gy fractions over 4 or 4.5 weeks, respectively and normal diet; (2) sham irradiation and normal diet; (3) WBI plus Pio (120 ppm) prior, during and for 4 or 54 weeks postirradiation; (4) sham irradiation and Pio diet; (5) WBI plus Pio starting 24 hours after completion of WBI. This study found that administering Pio prior to, during, and up to 4- or 54- weeks post-WBI significantly mitigated the WBI-induced cognitive impairment as measured by the object recognition test. However, the mechanism(s) involved in the radiation protection by PPAR*γ* is not known at present. 

 With reference to PPAR*α*, although we demonstrated that fenofibrate prevented the detrimental effect of WBI on hippocampal neurogenesis and inhibited microglial activation, we were unable to use the mouse model to test whether it can inhibit radiation-induced cognitive impairment. These mice have a 129/sv background and perform poorly in cognitive function tasks due to defects in their corpus callosum [119]. Currently we are using the existing rat model to investigate whether fenofibrate mitigates radiation-induced cognitive impairment. At present, the role of PPAR*δ* in radiation-induced brain injury is not known and is actively being investigated in our laboratory.

## 9. Putative Mechanism(s) of PPARs in Radiation-Induced Brain Injury

Both clinical and experimental evidence point out that the radiation-induced cognitive impairment can occur in the absence of gross histopathological and radiographical changes alterations [73–75]. These data suggest that more subtle cellular/molecular and functional changes (glial activation, neural precursor, endothelial and neuronal dysfunction) as result of increased inflammation/oxidative stress might play a role in the pathogenesis of radiation-induced late effects. 

**Figure 1:**
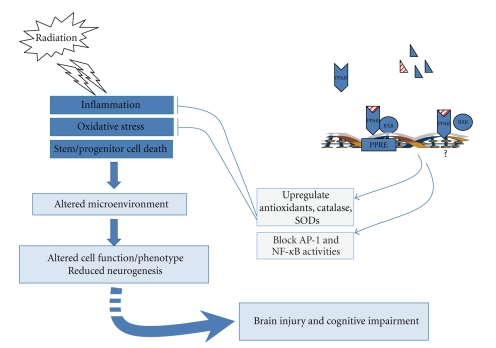
Model for the role of PPARs in radiation-induced brain injury. Irradiation is hypothesized to modify the brain microenvironment via the generation of an inflammatory and/or oxidative stress response which is also characterized by increased cell death of the neural precursor cells residing in the neurogenic regions of the brain. This alteration in the microenvironment is proposed to play a role in the dysfunction of the various cell-types in the brain (e.g., astrocytes, endothelial cells, microglia, neurons, and oligodendrocytes) and the reduction in ongoing adult neurogenesis ultimately contributing to radiation-induced brain injury including cognitive impairment. Activation of PPARs using specific ligands is hypothesized to play a role in normalizing the brain microenvironment and preserving cellular function following irradiation in part via inhibition of proinflammatory signaling pathways and by upregulation of antioxidant enzyme activities thus ameliorating the detrimental effects of radiation on the brain.

 Based on our findings, we propose a model for the role of PPARs in the regulation of radiation-induced brain injury. Irradiating the brain leads to increased proinflammatory response as evidenced by (1) increased activity of NF-*κ*B and AP-1 and (2) increased levels of TNF*α*, IL-1*β* and Cox-2. Microglia probably are the primary source of these mediators, although other cells likely contribute to the proinflammatory response [107, 120]. These cytokines might diffuse into the extracellular space and act on astrocytes, endothelial cells, neurons, and neighboring microglia, initiating a cytokine signaling cascade that alters the brain microenvironment (enhanced neuroinflammation, decreased hippocampal neurogenesis) and ultimately contributes to radiation-induced cognitive impairment (Figure 1). 

 While the exact role of these proinflammatory mediators in the pathogenesis of radiation-induced brain injury is currently under investigation, a clue to their function is suggested by studies with other brain injury models. Although required for the normal brain development, NF-*κ*B and AP-1 have been shown to be dysegulated in a number of CNS disorders. An up-regulation of c-jun activity has been observed following neuronal injury [121]. In addition, mutating the activating phosphorylation sites on c-jun protects against neuronal apoptosis in the hippocampus [122]. Similarly, in an experimental spinal cord injury model, the p65 subunit of NF-*κ*B was chronically activated in the microglia, endothelial cells and neurons adjacent to the lesion [123]. Increased levels of proinflammatory cytokines have been associated with a number of neuroinflammatory conditions such as Alzheimer's disease [124], Parkinson's disease [125], and multiple sclerosis [126]. Experimental augmentation of TNF*α*, IL-1*β*, and Cox-2 levels has been shown to induce behavioral and memory impairments in rodents [127–129]. TNF*α* and IL-1*β* have been shown to be potent inducers of apoptosis in oligodendrocytes and neural progenitor cells [130–132]. These data suggest that the elevated levels of proinflammatory mediators in the brain following irradiation could contribute to the pathogenesis of radiation-induced brain injury. 

 In a number of model systems, activation of PPARs has been shown to downregulate the expression of proinflammatory mediators, such as TNF*α*, IL-1*β*, Cox-2, and iNOS by interfering with the activity of transcription factors NF-*κ*B, AP-1, and STAT-1 (extensively reviewed in [2, 32]). Moreover, PPAR ligands have been shown to maintain the redox balance by upregulating the expression and activity of several antioxidant enzymes. A putative PPRE has been identified in the rat catalase promoter, the activity and expression of which was induced upon PPAR*γ* ligand treatment [133]. PPAR*α* and *γ* ligands downregulated phorbol ester-induced expression of NAPDH oxidase subunits p22^phox^ (message level) and p47^phox^ (protein levels), which was accompanied by increased superoxide dismutase (SOD) activity [134, 135]. PPAR*δ* agonists have been shown to inhibit TNF*α*-induced ROS generation by upregulating expression of antioxidant enzymes catalase, SOD-1, and thioredoxin in human umbilical vein endothelial cells [136]. Moreover, as described previously, PPAR ligands can modulate the severity of several CNS disorders. These data, along with our findings, support the hypothesis that activation of PPARs, via anti-inflammatory and/or antioxidant mechanisms, normalizes the brain microenvironment (characterized by reduced glial activation and preserved hippocampal neurogenesis) following WBI contributing to the amelioration of cognitive impairment following irradiation. 

 From our model, it is important to appreciate that altered hippocampal neurogenesis and microglial activation following WBI account for only one aspect of the pathogenesis of radiation-induced brain injury. The radiation response of the brain is complex and involves multiple pathways turned on in multiple cell types via autocrine, paracrine, and juxtacrine signaling mechanisms. Therefore, the relative contribution of other brain cells–astrocytes, endothelial cells, neurons and oligodendrocytes– to radiation injury cannot be excluded. In the future, investigating the radiation response of these cells and whether PPAR ligands can prevent their cellular dysfunction following irradiation will help shed more light on the mechanism(s) of radiation-induced brain injury and how it can be modulated.

## 10. Conclusions

Of the ~200 000 patients who receive WBI each year in the USA, up to 50% of them will develop progressive, cognitive impairment. There are no long-term treatment or prevention strategies for this debilitating side effect. Although the exact mechanisms remain ill-defined, increasing experimental evidence suggests a role for inflammation and/or oxidative stress during the pathogenesis of radiation-induced brain injury. PPAR ligands, given their propensity to target and modulate multiple proinflammatory pathways and their ability to upregulate antioxidant enzymes, appear to be an effective therapeutic strategy to modulate late effects following WBI. PPAR*α* ligands inhibit the radiation-induced proinflammatory responses in the microglia in vitro and prevent the detrimental effect of WBI on hippocampal neurogenesis in vivo. Moreover, the PPAR*γ* ligand Pio mitigated the WBI-induced cognitive impairment. Most importantly, PPAR*α* and *γ* ligands are FDA-approved and are routinely prescribed for the treatment of several chronic disorders such as hypertriglyceridemia, dyslipidemia, and Type 2 diabetes [17, 137, 138]. PPAR*δ* agonists are currently in Phase II clinical trials for dyslipidemia [139]. Therefore, these compounds offer the promise of enhancing the quality of life and long-term survival of cancer patients receiving brain irradiation.
